# The False and the True: A Warning

**Published:** 1881-07

**Authors:** George H. Clark

**Affiliations:** Philadelphia


### THE FALSE AND THE TRUE; A WARNING.

George H. Clark, M. D., Philada.

"Resolved, that we have no sympathy in common with those physicians who would engraft on homœopathy the crude ideas and doses of allopathy or eclecticism, and we do not hold ourselves responsible for their fatal errors in theory, and failures in practice."

This from the declaration of this Association at its formation, and as a contribution going to illustrate the necessity of it, the following:

Lately there came under observation, a case of deep opacity of the cornea with this history: Five months ago, after leaving a heated room and on going into the intensely cold air of the street, inflammation of the eye set in, which was pronounced sclerotitis by the attending homœpathic (?) physician, for which he used locally astringents, with internal medicines, two at a time, in alternation. The health of the gentleman in other respects was all that could be desired; yet here was a man in the prime of life, with one eye useless, and beyond the peradventure of a doubt, the treatment he received was the cause of his loss.

Had his attendant known the real nature of the trouble (keratitis), had he been even slightly familiar with the allopathic mode of treatment which he bunglingly attempted to follow, he would have known that in applying astringents, he was doing just what it is necessary to avoid in keratitis—for there is nothing better calculated to cause deep opacity, and even perforation, of the cornea, than astringents; and had he consulted any old school treatise on the subject, he could have learned as much. Had he even attempted to apply what little knowledge of homœopathy he possessed, there would now be one more serviceable eye in the community, and one more adherent of homœopathy.

At the time I met with this case, I had under treatment a lady, aged seventy-three years, suffering with keratitis of the left eye. She was, and had long been a sufferer from chronic arthritis; yet, notwithstanding one could not look for a most favorable result, vision in that eye is as good as before the attack: there is neither opacity or nebula.

These symptoms were at first present (in this, as in every other disease, names need not enter into the problem of remedy; rigid adherence to the rules laid down by HAHNEMANN will command success where success is possible; turning from the law is to flounder in the dark, and to be without a guide): Intense photophobia; lachrymation, tears, biting, burning; ciliary pain, worse from light; the characteristic zone of vessels about the margin, with small twigs extending across and toward the centre, and haziness of the cornea; a deep mist appeared before the eye, and in a few days this had so much increased that fingers could not be counted. *Euphrasia* 500, in water, a spoonful every four hours, was given for two days. Lachrymation and pain were much better at the end of that time, but the opacity seemed more dense.

*Sac-lac.* was often given and continued for one week, when the opacity appeared to be lessening; lachrymation almost gone; still some *photophobia.* One dose *Euphrasia* 500. In another week there was no photophobia, except in sunlight; the opacity was almost entirely gone, and she was able to read Jæger No. 7 at the usual distance with her glasses. This was all, and the only adjuvant used was a shade for the affected eye—and, has been remarked, vision in that eye is normal. Compare these, and then ask if homœopathy can do all that is desirable in such cases. In the one case, a man in apparently perfect health is attacked with inflammation of the cornea; is treated, as he thought, homœpathically; result, loss of vision. In the other, a woman beyond the allotted three-score and ten, with a chronic disease that impaired to a great extent her recuperative powers, has a similar affection, and with the simple, grand homœopathic treatment as outlined, and filled in by Hahnemann, has her sight preserved to her, and is able to call down blessings on the master.

"Example is better than precept." Respect for, and an imitation of what is known to be true, can more readily be gained from those who differ, by one's closely following what experience has taught to be the best mode of procedure in anything; and the result will be more convincing. To him who is engaged in doing the best that can be done for disease, many cases that have been treated by others who profess to follow the course he follows, often appear, and present conditions that merit and call forth his hearty contempt. Instead of temporizing with the homœopathic law, as is constantly done in so-called homœopathic colleges, every student must be taught, and every practitioner have constantly before him, the prime necessity of adhering rigidly to that law.

